# Antimicrobial resistance: capacity and practices among clinical laboratories in Kenya, 2013

**DOI:** 10.11604/pamj.2014.19.332.5159

**Published:** 2014-11-27

**Authors:** Fredrick Odhiambo, Tura Galgalo, Arvelo Wences, Onesmus Maina Muchemi, Evalyne Wambui Kanyina, Juliana Chepkemoi Tonui, Samwel Amwayi, Waqo Boru

**Affiliations:** 1 Field Epidemiology and Laboratory Training Program, Kenya; 2 Ministry of Health-Kenya, Kenya

**Keywords:** Capacity, antimicrobial resistance, isolates, culture, susceptibility, practice, pattern

## Abstract

**Introduction:**

Antimicrobial resistance is neglected in developing countries; associated with limited surveillance and unregulated use of antimicrobials. Consequently, delayed patient recoveries, deaths and further antimicrobial resistance occur. Recent gastroenteritis outbreak at a children's home associated with multidrug resistant non-typhoidal *Salmonella* spp, raised concerns about the magnitude of the problem in Kenya, prompting antimicrobial resistance assessment preceding surveillance system establishment.

**Methods:**

Eight public medical laboratories were conveniently selected. Questionnaires were administered to key informants to evaluate capacity, practice and utilization of antimicrobial susceptibility tests. Retrospective review of laboratory records determined antimicrobial resistance to isolates. Antimicrobial resistance was defined as resistance of a microorganism to an antimicrobial agent to which it was previously sensitive and multidrug resistance as non-susceptibility to at least one agent in three or more antimicrobial categories.

**Results:**

The laboratories comprised; 2(25%) national, 4(50%) sub-national and 2(25%) district. Overall, antimicrobial susceptibility testing capacity was inadequate in all. Seven (88%) had basic capacity for stool cultures, 3(38%) had capacity for blood culture. Resistance to enteric organisms was observed with the following and other commonly prescribed antimicrobials, ampicillin: 40(91%) *Salmonella* spp isolates; Tetracycline: 16(84%) *Shigella flexineri* isolates; cotrimoxazole: 20(100%) *Shigella* spp isolates, 24(91%) *Salmonella* spp isolates. Comparable patterns of multidrug resistance were evident with *Shigella* flexineri and *Salmonella typhimurium*. Ten (100%) clinicians reported not using laboratory results for patient management, for various reasons.

## Introduction

The problem of antimicrobial resistance (AMR) is major in resource-limited countries, mostly because of poverty [1, 2] and lack of proper surveillance systems. Surveillance of AMR generally provides data that is needed to raise the awareness of the problem and to implement necessary interventions [3]. Principally, laboratory-based surveillance is required for detection of resistance and monitoring for its spread [4]. Therefore, AMR patterns could very well be related to medical laboratories’ ability and practices in performance of cultures and antimicrobial susceptibility testing (AST), and rationality of prescriptions by clinicians [3, 4]. In view of these, developing countries are being called upon to improve access to diagnostic laboratories, institute surveillance of emergence of resistance and regulate use of antibiotics besides instituting comprehensive national AMR policies and strategies [3]. Similarly, the increasing interconnection between countries and the globalization of trade and travel promotes the risk of importing bacteria or genes that could lead to the spread of AMR across borders and jeopardize effective treatment or prevention of bacterial infections [5]. Antimicrobial resistance being an emerging public health threat with local, national and global dimension has consequences ranging from delays in recovery to deaths from infectious diseases [6, 7] and further spread of resistance. As such, resistance to first-line drugs in most of the pathogens ranges from zero to almost 100% [4].

In Kenya, a recent outbreak of gastroenteritis at Mama Ngina Childrens’ Home in December 2012 where multi- drug resistant (MDR) strain of non-typhoidal *Salmonella* spp was reported, in which two deaths occurred raised concerns about the magnitude of the problem in the country. This prompted the Ministry of Public Health and Sanitation to conduct rapid assessment to establish antimicrobial susceptibility testing capacity, practices and antimicrobial resistance patterns in selected clinical and public health laboratories. In determining the most appropriate indicator for AMR in the events that led to this study, focus was on enteric pathogens. Moreover, diarrhoeal diseases are a major cause of morbidity and mortality in developing countries, where bacteria is the most important pathogens in older children and adults, because of rampant empirical treatments in part contributed by inadequacies of laboratory services [8]. Consequently, the spread and gradual replacement of drug-sensitive strains of *Salmonella typhi* with multidrug-resistant strains that threatens to reduce clinical options for treating typhoid fever have been reported [9]. Evidence is also available from a study at Kenyatta National Hospital (KNH) indicating that the prevalence of *Salmonella typhi* resistant to two or more antimicrobials has been on the rise; from 50% in 1998 to 78% in 2004 [10]. These patterns are also likely to be observed with other enteric bacterial pathogens. In a bid to establish in-depth account of AMR and MDR in Kenya following the stated events, a rapid assessment was conducted in government owned medical laboratories.

## Methods

**Study sites:** eight public medical laboratories consisting of two level 6, four level 5 and two level 4 health facilities were conveniently selected. Level 6 was defined as national referral facility, level 5 as sub-national facility and level 4 as district facility. These facilities were targeted because of being high volume laboratories with better resources for bacterial culture and AST, therefore considered to be better placed in reporting AMR as well as MDR.

**Study design:** rapid assessment; review of medical laboratory records for one year and assessment of laboratory capacity in AST using semi-standardized forms and check list. Key informant interviews (KII) were conducted by interviewing key laboratory personnel and clinicians in the facilities.

**Definitions of antimicrobial resistance (AMR):** antimicrobial resistance was defined as resistance of a microorganism to an antimicrobial medicine to which it was previously sensitive [11]. On the other hand, MDR was defined as acquired non-susceptibility to at least one agent in three or more antimicrobial categories [12].

**Data collection:** retrospective reviews of laboratory records on AST for stool and blood cultures were carried out alongside KII. Data on culture and AST from January to December 2012 was extracted from bacteriology registers using semi-standardized forms. This was aimed at describing enteric isolates and determining AMR patterns. Key informant interviews using semi-structured questionnaire for which all affirmative responses would be verified were administered to bacteriology section-heads of the respective medical laboratories and hospital clinicians. This was to assess capacity of the laboratories to perform culture and AST, culture and AST practices, and utilization of the laboratory culture and AST results by clinicians. Data on culture media, reagents, typing sera, equipment, stock organisms, infrastructure, quality assurance, archiving systems, outbreak preparedness, use of Clinical and Laboratory Standards Institute (CLSI) or related standards, turnaround time, and categories of antibiotic discs used were collected in the process.

**Data management and analysis:** data from responses on KIIs and review of bacteriology records was cleaned, entered in Epi Info and Microsoft Excel 2010 spread sheets from which counts, frequencies and tables were obtained. Aggregation and data summaries were made for all laboratories assessed.

**Ethical consideration:** the permission to carry out the assessment was granted by the Ministry of Health (MOH) and the medical superintendents of the respective health facilities where laboratories were located. To maintain confidentiality, no patient identification information was extracted from the registers during the review process. The findings were disseminated in a breakfast meeting attended by MOH and line ministry officials among other stakeholders. Reports of the findings were sent to all participating health facilities and other stakeholders. The findings have also been presented in two international conferences; the 5th African Field Epidemiology Network Scientific Conference and the 2nd joint Infection Prevention Network Kenya (IPNET-K)/ Infection Control Africa Network (ICAN) conference.

## Results

**Facility description:** between 10^th^ and 28^th^ February 2013, we assessed eight medical laboratories and analyzed data. All participating facilities were high volume, public medical laboratories. They comprised 7(88%) clinical and 1(13%) public health laboratories, and all except one were participating in WHO/AFRO stepwise laboratory improvement scheme.

**Laboratory capacity:** all laboratories had basic capability to perform bacterial culture and sensitivity tests for common bacterial pathogens; however, only 1(13%) laboratory had facilities for isolation of *Campylobacter* spp although there were no reports of this organism being isolated. Despite the availability of basic capacity to perform culture and sensitivity testing, 1(13%) laboratory did not have any records and only 3(38%) performed blood cultures. Two (25%) facilities had high throughput automated equipment (Bactec™ machine) for blood culture; however, both reported not performing blood cultures. All (100%) laboratories reported not having service contracts for bacteriology equipment and only 1(13%) had equipment validation reports. Six (75%) laboratories reported having inadequate staff and lack of specialists in bacteriology and only 2(25%) laboratories reported that all their staff had successfully undertaken competency tests in culture and AST at the time of assessment. All the technicians from 7(88%) of the laboratories reported not having undergone any refresher training in microbiology techniques in the past one year. However, all technicians from 1(13%) of the national reference laboratories reportedly participate in an in-house continuous professional development sessions.

Seven (88%) laboratories assessed had adequate optional biochemical tests (bio typing) for bacteriology culture identification. These optional tests do not expressly identify microorganism, and more specific tests are required for this. Six (75%) laboratories had sufficient stocks of Thiosulfate Citrate Bile Salts sucrose agar (TCBS) and Alkaline Peptone Water (APW) essential for isolation of *Vibrio Cholerae*.

**AST practices:** overall, bacterial culture and AST practices varied in all the eight laboratories assessed. There were 5(63%) laboratories with stool sample collection standard operating procedures (SOPs), 7(88%) with culture processing SOPs and 5(63%) with AST SOPs. All (100%) laboratories had daily maintenance reports for the essential microbiology equipments; however, 2(25%) laboratories reported use of faulty microscopes. Installation reports of basic bacteriology equipment were available in 5(63%) facilities. Varied blood culture bottles were used in all the 3 laboratories performing blood culture. Each of the 3(38%) laboratories that performed blood culture used different primary culture media, with one using diphasic-Thioglycolate, another using diphasic- Haemolysin and another using Brain-heart infusion. Blood sub-culture media in one national referral laboratory comprised only of Chocolate Blood Agar (CBA) and Cystine Lactose Electrolyte Deficient Agar (CLED). Laboratories are required to ensure standard depth of media during culture, but only 1(13%) reported use of calibrated media dispenser. Selenite F broth is an enrichment media essential for *Salmonella* spp isolation in stool, but only 3(38%) of the laboratories were using it (Table 1). Stock of expired stool culture media and reagents were observed in two laboratories.

**Table 1:** Description, practice and capacity of health facilities selected for AMR assessment in Kenya, 2013

Item	n (%)
**Facility category**	
level 6	2(25)
Level 5	4(50)
Level 4	2(25)
**Presence of SOP**	
specimen collection	5(63)
culture processing	7(88)
AST	5(63)
**Media and Reagents**	
Transport Media avail.	5(63)
Media Prep. Room	6(75)
stock TCBS and APW	6(75)
use of McFarland	5(63)
**Typing sera avail**	
V. cholera	6(75)
E. coli	0(0)
Shigella	2(25)
Salmonella	4(50)
**Quality Assurance**	
Archiving system avail	1(13)
TAT sample processing (2hrs)	4(50)
Service contract avail	0(0)
adherence to CLSI	2(25)
**Barriers to clinician AST utilization**	
Not using lab. Results	8(80)
Delay of results	3(30)
No feedback	5(50)
Limited Lab operation hrs	2(20)
AST drug absent in hosp. pharm	10(100)

Note: AST, Antimicrobial susceptibility testing; n, Frequency;%, Percent; Prep, Preparation; TCBS, Thiosulfate Citrate Bile Salts sucrose agar; APW, Alkaline peptone water; Avail, Available; V. cholerae, *Vibrio cholerae*; E.coli, *Escherichia coli*; TAT, Turnaround time; hrs., hours; CLSI, Clinical and laboratory standards institute; lab., laboratory; hosp., hospital; Pharm, Pharmacy

Five (63%) facilities performed Internal Quality Controls (IQC) on media and reagents, while 3(38%) participated in microbiology External Quality Assurance (EQA), though not for culture and AST. Two (25%) laboratories consistently used Mueller Hinton media for AST, 3(38%) used single discs for AST, while 5 (63%) reported use of Mc Farland standard and stocked standard organisms for quality control (Table 1). Overall, none of the assessed facilities had capability to characterize and identify pathogenic *Escherichia coli*, although reports of the organism were obtained in 4 (50%) of them. Six (75%) laboratories had typing sera for *Vibrio cholera*, 4 (50%) had typing sera for *Salmonella* spp and 2 (25%) had tying sera for *Shigella* spp. *Salmonella* and *Shigella* species were however reported by 7 (88%) laboratories without evidence of specific species serotyping (Table 1).

**Enteric bacteria isolation:** four thousand nine-ninety seven (4997) stool cultures were reported compared to blood's 4258. Of these, bacterial agents were isolated in 827(17%) stool cultures and in 70(2%) blood cultures (Table 2). The median detection rate of enteric pathogens in stool in the eight facilities was 3% (range= 1-19%) while the mean detection rate in blood was 2% (SD = 1) (Table 3). Cultures in which no pathogens were isolated amounted to 4170 (84%) for stool and 4188 (98%) for blood. *Escherichia coli* were the highest isolated enteric bacterial agent, obtained in 617(12%) stool cultures and 31(1%) blood cultures. On average, 75(2%) stool cultures obtained *Salmonella* species isolates, while 105(2%) stool cultures obtained *Shigella* species isolates. Non-pathogenic enteric bacterial agents (Klebsiella spp, Citrobacter spp, Proteus spp and *Pseudomonas* spp) were also isolated and reported in 34(1%) stool cultures. In blood, *Salmonella* species was the second most isolated agent, obtained in 19(*Pseudomonas* spp in 2(*Streptococcus pyogenes* in 1(Table 2).

**Table 2:** Enteric bacterial agents isolated from stool and blood culture as reported in the eight assessed laboratories in Kenya, 2013

	Stool	Blood
Organism	n (%)	n (%)
No pathogen	4170(84)	4188(98)
E.coli	617(12)	31(<1)
**Salmonella**		
*Salmonella* spp	49(<1)	12(<1)
*Salmonella typhi*	13(<1)	7(<1)
*Salmonella paratyphi*	3(<1)	0
**Shigella**		
*Shigella* spp	34(<1)	0
*Shigella boydii*	24(<1)	0
*Shigella flexineri*	30(<1)	0
*Shigella sonnei*	5(<1)	0
*Shigella dysentriae*	12(<1)	0
**Other bact. Agents**		
*Staphylococcus* SPP	0	7(<1)
*Pseudomonas* spp	0	2(<1)
*Proteus* spp	0	1(<1)
*Strept. pyogenes*	0	1(<1)
Others	34(<1)	0
**Total**	**4997(100)**	**4258(100)**

Note: n, Frequency; E. coli, *Escherichia coli*; spp, species; bact., bacterial; Strept, *Streptococcus*; <, less than

**Table 3:** Isolation rates of enteric pathogenic bacteria agents in the eight assessed laboratories in Kenya, 2013

	Stool culture			Blood culture		
Facility	No. tested	No. detected	Detection Rate	No. tested	No. detected	Detection Rate
Nat1	1,970	16	1%	3907	58	2%
Nat2	589	50	9%	0	0	NT
Sub-Nat1	534	16	3%	42	1	2%
Sub-Nat2	191	5	3%	309	10	3%
Sub-Nat3	951	13	1%	0	0	NT
Sub-Nat4	689	62	9%	0	0	NT
District1	73	14	19%	0	0	NT
District2	0	0	NT	0	0	0
**Total**	4,997	176	4%	4258	69	2%

Note: No, number; Nat, National; NT, Not tested; Pathogenic bacteria agents, *Salmonella* and *Shigella* species

**Antimicrobial resistance patterns:** The following organisms showed AMR patterns to ampicillin: 8(80%) *Shigella dysenteriae* isolates, 3(75%) *Shigella sonnei* isolates, 7(78%) *Shigella boydii* isolates and 24(90%) *Salmonella* spp isolates. Similar resistance patterns were observed with tetracycline, in which16 (84%) *Shigella flexineri* isolates, 5(71%) *Shigella boydii* isolates and 9(76%) *Salmonella* spp were obtained (Table 4). Resistance was observed with 5(100%) *Shigella* spp. isolates to cotrimoxazole. Fifteen (91%) *Salmonella* spp. isolates also showed marked resistance to cotrimoxazole. Similarly, *Salmonella* spp isolates showed relatively high resistance to sulfamethoxazole, while absolute resistance to the same antimicrobial agent was observed with 7(100%) isolates of *Shigella dysentriae*, 4(100%) *Shigella sonnei*, 7(100%) *Shigella boydii* and 3(100%) *Shigella flexineri*. Two (100%) isolates of *Salmonella* spp and 2(100%) of *Shigella flexineri* showed absolute resistance to amoxicillin. High resistance to amoxicillin was also observed with 7(78%) isolates of *Shigella boydii*. Twenty nine (90%) *Escherichia coli* isolates were also resistant to ampicillin, 3(60%) to cotrimoxazole and 21(60%) to cefotaxime. Multidrug resistance according to the definition adopted in this study, might have occurred in cultures of *Shigella flexineri* and *Salmonella typhimurium* isolates. *Shigella flexineri* exhibited MDR tendencies where 3(100%) were resistant to cotrimoxazole, 3(100%) to sulfamethoxazole and 2(100%) to amoxicillin. *Salmonella* typhimurium on the other hand exhibited MDR, with 6(100%) being resistant to ampicillin, 2(100%) to cotrimoxazole and 3(100%) to cefotaxime. However, it could not be ascertained whether the same isolates of the two species were subjected across the three antimicrobial agents (Table 4).

**Table 4:** Resistance patterns of enteric pathogens in stool and blood in the eight assessed laboratories to commonly prescribed antimicrobials in Kenya, 2013

Resistance							
Organism	Amp	Tet	Cot	Aug	SMZ	Amox	Cefot
n (%)		n (%)	n (%)	n (%)	n (%)	n (%)	n (%)
*Salmonella* spp	24(90)	9(76)	15(91)	3(100)	8(73)	2(100)	2(50)
*Salmonella typhi*	10(82)	2(33)	7(87)	0(0)	3(75)	0(0)	1(17)
*Salmonella typhimurium*	6(100)	NT	2(100)	NT	NT	NT	3(100)
*Shigella* spp	0(0)	0(0)	5(100)	5(63)	1(100)	5(83.3)	NT
*Shigella dysentriae*	8(80)	5(63)	2(100)	NT	7(100)	NT	NT
*Shigella sonnei*	3(75)	2(67)	NT	NT	4(100)	NT	NT
*Shigella boydii*	7(78)	5(71)	10(100)	3(75)	7(100)	4(80)	NT
*Shigella flexineri*	8(36)	16(84)	3(100)	1(50)	3(100)	2(100)	NT
*E. coli*	26(90)	NT	3(60)	NT	NT	NT	21(60)

Note: n, Frequency; AMP, Ampicillin; Tet, Tetracycline; Cot, Cotrimoxazole; Aug, Augmentin; SMZ, Sulfamethoxazole; Amox, Amoxicillin; Cefot, Cefotaxime; spp, species; NT, Not tested

**AST utilization:** Ten clinicians were interviewed on utilization of AST results for patient management. Eight (80%) of the clinicians reported not utilizing laboratory AST results for patient management despite having knowledge of the services being available at their hospital laboratories. The reasons for underutilization of AST services by clinicians varied; all (100%) indicated that antibiotics tested by the laboratories were not available in their hospital pharmacies. Three (30%) blamed delays in AST laboratory results, 5(50%) blamed lack of feedback from the laboratory and 2(20%) cited limited laboratory operation hours as the barriers to their utilization of culture and AST services (Table 1).

## Discussion

In the study to assess AMR, AST capacity and practices of medical laboratories, we found that commonly prescribed first-line antimicrobial agents are facing threat of resistance to common enteric pathogens in Kenya. Capacity of bacterial culture and AST in the assessed medical laboratories was inadequate. This undermines the quality and validity of results obtained from the laboratories. The available equipment for example, in all the laboratories assessed lacked validation reports and most lacked service contracts, posing a major quality challenge. Competence inadequacies were also evidenced by most laboratories reporting *Escherichia coli*, *Salmonella* and *Shigella* species without any evidence of serotyping.

Lack of use of certain essential media was also noted, with only three laboratories reporting use of Selenite F broth for isolation of *Salmonella* spp in stool culture, which reflected the inability of the other laboratories to isolate *Salmonella* spp especially when they are in low numbers. Mueller Hinton media and use of Mac Farland are very critical in AST among other quality processes. However, five of the eight laboratories used Mac Farland standards while only two consistently used Mueller Hinton media for AST. Stock of expired stool culture media and reagents were observed in two laboratories. Expired laboratory reagents when not removed from the shelves and disposed appropriately might tempt technicians to use them especially when experiencing stock outs. Use of expired reagents for testing leads to incorrect results. Cholera outbreak preparedness was evident in six facilities which had stock of both TCBS media and APW and five facilities which had stock transport media for sample transfer from the field. Five of the eight laboratories had stocked standard organisms for quality control. The rest of the laboratories could not effectively assure quality of the various culture media and reagents for lack of standard organisms.

We also found that bacterial culture practices were varied across the participating laboratories. For instance, blood culture bottles varied from use of conventional blood culture bottles as observed in one of the national level and one sub-national level laboratory to improvised blood culture bottles in another sub-national level laboratory. Improvised blood culture bottles might not be sterile thus a potential source of media contamination leading to false positive results and overall compromising quality of laboratory outcomes. Some laboratories lacked important SOPs for stool culture and AST. SOPs aims at standardizing the operations within the laboratory. Lack of approved SOPs for laboratory procedures will compromise the reliability and accuracy of the results performed by different technicians [13]. Other practices, as use of varied primary and sub-culture media for blood culture observed in the assessed laboratories, indicate lack of clear standards and guidelines in place. Clinical and Laboratory Standards Institute guidelines for AST were also only used in national level facilities; other laboratories lacked any standardized guidelines for antimicrobial susceptibility testing. A wide range of antibiotics was subjected to AST across most laboratories without reference to any approved guideline. This finding is inconsistent with the ideal practice in microbiology which defines that AST should be done according to CLSI [14] or any other approved guideline.

The results indicated that proportion of stool cultures with enteric bacterial pathogens isolated was 3.5%. This isolation rate is unlikely to be a true reflection of the distribution and occurrence of the pathogenic enteric bacteria in the population. During a 1-year period surveillance study in Western Kenya in which 729 stool specimens were collected from patients with diarrhea, 244 (33%) of the specimens yielded *Shigella*, Campylobacter, *Salmonella*, or *Vibrio* species [15]. This clearly indicates that the isolation rate of enteric pathogens in the assessed sites was low, occasioning concerns about laboratory capacity, practices and to some extent suspicion index by clinicians. Lack of enrichment media for stool cultures in four of the laboratories may have in part contributed to poor isolation rate. Other factors which might have contributed to low isolation rate of enteric bacteria by laboratories were: use of expired media and reagents, lack of internal quality controls (IQC) and standard organisms and poor processes in the pre-analytical phase like improper specimen collection, transport and delay in stool culture. However, AMR patterns, particularly of *Salmonella typhimurium* to cotrimoxazole, cefotaxime and ampicillin was consistent with those of other non-typhoidal *Salmonella*s described in Kenya by Kariuki et *al* [16].

The current AST practices as described in this document are largely resource wasting, undesirable in a resource-limited country, like Kenya. This assertion is supported by the fact that majority of the clinicians interviewed do not use laboratory AST results for patient management due barriers that can be avoided. **Limitations of the assessment:** This study had a number of limitations which included convenient rather than random selection of health facilities, which essentially limits representativeness and generalizability. Other limitations include incompleteness of microbiology registers which made it difficult to conclusively measure AMR and MDR. Lack of standard practice in AST further complicated the ability to effectively compare antimicrobial agents in terms of resistance to the isolates, since there was no uniformity in antimicrobial agents subjected to specific organisms. Further, due to inadequacies in standards of isolation and characterization of bacterial agents from both stool and blood, it was not possible to authenticate the various isolates. Despite these limitations, the assessment may well represent the Kenyan situation in terms of antimicrobial resistance.

## Conclusion

The basic equipment for AST which include microscopes, incubators, fridges and autoclaves were available in all the assessed laboratories. However, the practices and capacity for all these laboratories to perform bacterial culture and AST was deficient, which may support the observed low isolation rates. To a great extent, there were no approved standards being used in culture and AST, hence the patterns observed with the bacterial isolates and AMR/MDR may not be conclusive. However, the observations made may be a pointer to potential microbial resistance with commonly prescribed antimicrobials. **Recommendations:** there is need to establish and strengthen capacity of microbiology laboratories in terms of technical skills, staffing, documents, mentorship, infrastructure, procurement, supplies (reagents, materials) and equipment. There is need to standardize culture and antimicrobial susceptibility testing methods in medical laboratories in Kenya. It is necessary to establish antimicrobial resistance surveillance system for common enteric pathogens. There is need for study on policy, standards, guidelines and regulation to ensure appropriate antimicrobials use.
